# 

**Published:** 1874-06

**Authors:** 


Vol. IV. JUNE, 1874. No. 6.

### THE SOUTHERN MEDICAL RECORD:

A MONTHLY JOURNAL OF

### PRACTICAL MEDICINE.

EDITORS:

| | |
|---|---|
| T. S. POWELL, M.D., | Atlanta, Georgia. |
| W. T. GOLDSMITH, M,D., | Atlanta, Georgia. |
| T. CURTIS SMITH, M.D., | Middleport, Ohio. |
| SWAN M. BURNETT, M.D., | Knoxville, Tennessee. |
| ALBAN S. PAYNE, M.D., | Markham's, Virginia. |
| A. R. KILPATRICK, M.D., | Navasota, Texas. |
| A. A. LYON, M D., | Columbus, Mississippi. |
| R. C. WORD, M.D., | Decatur, Georgia. |

*"QUICQUID PRÆCIPIES ESTO BREVIS."*

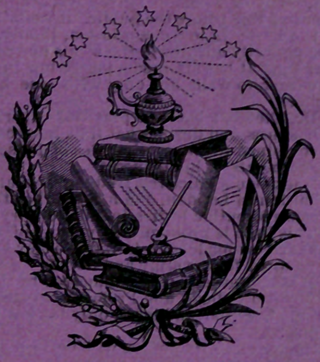

ATLANTA, GEORGIA:
SOUTHERN PUBLISHING COMPANY, PRINTERS.
1874.

Terms, $2 50 Per Annum, in Advance. Single Number, 25 Cents.

